# Automated classification of lay health articles using natural language processing: a case study on pregnancy health and postpartum depression

**DOI:** 10.3389/fpsyt.2023.1258887

**Published:** 2023-11-20

**Authors:** Braja Gopal Patra, Zhaoyi Sun, Zilin Cheng, Praneet Kasi Reddy Jagadeesh Kumar, Abdullah Altammami, Yiyang Liu, Rochelle Joly, Caroline Jedlicka, Diana Delgado, Jyotishman Pathak, Yifan Peng, Yiye Zhang

**Affiliations:** ^1^Department of Population Health Sciences, Weill Cornell Medicine, New York, NY, United States; ^2^Department of Obstetrics and Gynecology, Weill Cornell Medicine, New York, NY, United States; ^3^Kingsborough Community College, City University of New York, New York, NY, United States; ^4^Samuel J. Wood Library & C.V. Starr Biomedical Information Center, Weill Cornell Medicine, New York, NY, United States

**Keywords:** online health information, health communication, natural language processing, pregnancy, postpartum depression

## Abstract

**Objective:**

Evidence suggests that high-quality health education and effective communication within the framework of social support hold significant potential in preventing postpartum depression. Yet, developing trustworthy and engaging health education and communication materials requires extensive expertise and substantial resources. In light of this, we propose an innovative approach that involves leveraging natural language processing (NLP) to classify publicly accessible lay articles based on their relevance and subject matter to pregnancy and mental health.

**Materials and methods:**

We manually reviewed online lay articles from credible and medically validated sources to create a gold standard corpus. This manual review process categorized the articles based on their pertinence to pregnancy and related subtopics. To streamline and expand the classification procedure for relevance and topics, we employed advanced NLP models such as Random Forest, Bidirectional Encoder Representations from Transformers (BERT), and Generative Pre-trained Transformer model (gpt-3.5-turbo).

**Results:**

The gold standard corpus included 392 pregnancy-related articles. Our manual review process categorized the reading materials according to lifestyle factors associated with postpartum depression: *diet, exercise, mental health*, and *health literacy*. A BERT-based model performed best (F1 = 0.974) in an end-to-end classification of relevance and topics. In a two-step approach, given articles already classified as pregnancy-related, gpt-3.5-turbo performed best (F1 = 0.972) in classifying the above topics.

**Discussion:**

Utilizing NLP, we can guide patients to high-quality lay reading materials as cost-effective, readily available health education and communication sources. This approach allows us to scale the information delivery specifically to individuals, enhancing the relevance and impact of the materials provided.

## 1 Background and significance

Pregnancy is a vulnerable period that exposes patients to heightened anxiety, depression, and stress. One in seven birthing parents develops postpartum depression (PPD), a potentially life-threatening mental health condition and a much higher proportion of pregnant patients experience antenatal psychosocial stress (1, 2). Anxiety, depression, and stress lead to adverse maternal, infant, and family outcomes, disproportionately affecting disadvantaged families (1, 3). The negative impact can be mitigated by interventions from healthcare providers (4, 5). However, resource constraints, compounded with the sensitive nature of pregnancy and stigma against mental health, present a unique challenge in the prevention, screening, and management of mental health concerns in the clinical settings (1, 6). Disparities in the distance to healthcare, health literacy, socioeconomic status, and neighborhood characteristics further strangulate equitable access to clinical interventions during pregnancy (4, 7, 8).

In turn, patients resort to self-management to relieve stressors and resolve individual questions around pregnancy (9). Patients have grown to be active online information seekers (10–12), of which people of childbearing age are the most active members in the digital space in the US (13). Three-quarters of the US pregnant population is known to seek information about pregnancy health and birth online (14). However, a myriad of information and players with unspecified motives exist online, making it often difficult for lay audiences, particularly those with low health literacy, to comprehend and act appropriately (15, 16). Notably, the appropriateness of online content was identified as a barrier to user satisfaction and continued engagement among the pregnancy population (17, 18).

Our research is aimed to develop a platform to deliver personalized health education and communication materials. The Support Personalized prEgnancy Care with Artificial inteLligence (SPECIAL) platform (https://www.specialdayshealthinfo.com/) houses content on health education and communication developed by a commercial vendor. This study aimed to assess the feasibility of utilizing natural language processing (NLP) to repurpose publicly accessible lay reading materials from magazines and online sources as health education and communication content.

### 1.1 Related work

Numerous studies have explored strategies for evaluating online health information and utilizing it for health education, communication, and promotion (12, 19, 20). However, our approach in this study diverges from existing literature. We present an innovative method that streamlines the search for online lay articles and information based on relevance and topic, effectively alleviating the health literacy challenges and search burden experienced by patients. Studies most relevant to us are previous work in categorizing news from public datasets such as TagMyNews using various machine learning and optimization techniques (21). Likewise, there are studies on the classification of health-related information and news from social media, such as the extraction of information related to Zika virus, syndromic surveillance, and identifying misinformation from social media posts (22–25).

### 1.2 Objective

Extensive theoretical research and empirical evidence have consistently established the crucial role of health education and communication, particularly within the framework of social support, in determining health outcomes during pregnancy (9). As part of our ongoing study on preventing postpartum depression (PPD) through intervention development, we propose employing a machine learning approach to deliver health education and communication. This approach aims to assist patients in recognizing sources of support and enhancing their self-management abilities. Our objective is to develop an NLP model by curating relevant lay articles on pregnancy health that can serve as effective health education and communication materials for patients.

Our focus on lay articles stems from their accessibility and the ease with which patients can comprehend them, even without an advanced level of health literacy. In order to establish a scalable and sustainable approach, we hypothesize that NLP can be employed to identify current and credible reading materials pertaining to PPD from lay sources. By leveraging NLP techniques, we aim to provide patients with up-to-date and reliable resources related to PPD, thus enhancing their overall health literacy and empowering them to make informed decisions.

## 2 Materials and methods

### 2.1 Data construction

We identified articles from online sources such as Mayo Clinic, MedlinePlus, and American Pregnancy Association. We also collected medically reviewed articles from non-medical sources with easy accessibility and high recognition that are written with the lay population as the target audience. We looked for articles related to PPD risk factors and social support. Using Vaux's theory of social support (26), we centered the article classification around social support constructs and focused on articles and resources that serve as instrumental (diet, exercise), informational (health literacy), and emotional (wellness) support. Here, “informational” refers to articles that are related to pregnancy but do not fall into the previous three categories. We collected an initial set of articles by online search using keywords including *pregnancy diet, pregnancy exercise, pregnancy fitness, pregnancy yoga, pregnancy mental health, pregnancy anxiety, pregnancy mood swings, depression during pregnancy, mental wellbeing pregnancy, mental health AND pregnancy, pregnancy and mental issues, Perinatal mood and anxiety disorder (PMAD) in pregnancy, anxiety during pregnancy*, and *dealing with stress during pregnancy*. As we envisioned that the articles would be a preventative measure against PPD for pregnant readers, we intentionally excluded articles that mentioned PPD to avoid alerting patients to the potential disease risk (27).

Collected articles were then reviewed for selection following NIH recommendations on how to evaluate health information on the internet (28). The recommendations focused on evaluating the ownership, purpose, and funding source of the website hosting the information; the evidence used to support the information; reviewers of the information (medically reviewed or not); the year of publication; consumer information collected by the site, and availability of monitoring for online interactions among consumers. Based on these criteria, we prioritized articles that were medically reviewed or published by a non-profit organization to serve as the corpus of the pipeline. Our content validation process also included a credibility check on the collected articles to avoid misinformation and conflicting information across sources. This secondary process excluded articles that were published by non-US sources, were not written by certified specialists holding degrees such as M.D. and Ph.D., and were not published by websites well-known to the general public. Finally, we excluded articles on mixed topics that contained less than 80% of content related to pregnancy, and we also excluded articles published before 2019 to ensure the timeliness of the information.

**Figure 1:**
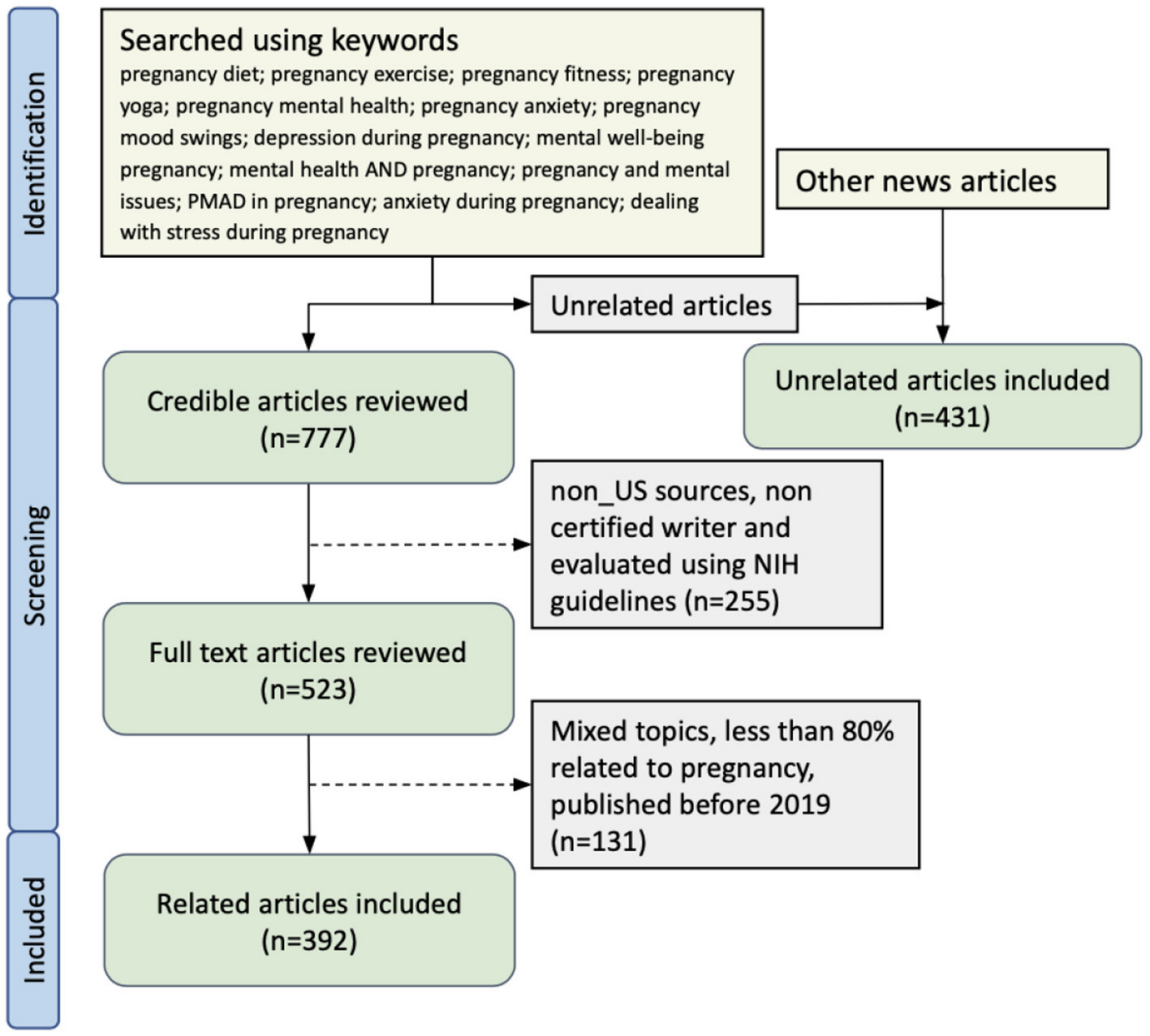
News articles inclusion criteria.

We identified 777 articles based on the above criteria from online searches. There were 523 credible articles left after the content validation step. Finally, we had 392 articles in our defined categories for training the NLP models. As a control, we also selected 431 articles that were not related to pregnancy. A list of unrelated news article topics is mentioned in Supplementary Table S5. The details of the article collection are shown in Figure 1 and counts of articles are provided in Table 1. Each article was reviewed by two annotators and the Cohen kappa metric for inter-rater reliability was 0.95. Any disagreement between reviewers was resolved by a third reviewer. During the review process, we identified the sources of the collected articles. Going forward, we will utilize these verified and authentic sources to gather pregnancy-related articles. To streamline the process, we have developed automated two web crawlers specifically tailored for each source, enabling efficient article collection.

**Table 1:** Characteristics of the dataset.

**Tag**	**Categories**	**N**	**Avg # words**	**Avg # sentences**
Related		392	1,231.44	59.41
	Diet	99	1,177.29	54.73
	Exercise	96	1,212.38	62.34
	Mental health	97	1,188.59	53.16
	Health literacy	100	1,347.48	67.41
Unrelated		437	1,343.51	61.37

### 2.2 Model development

We employed machine and deep-learning models to classify the collected articles. We concatenated the title and full-length text as the input. For both titles and full-length text, we removed stop words using the Natural Language Toolkit (NLTK) (29). We experimented with two approaches: an end-to-end approach and a two-step approach. The end-to-end approach treated articles as five topics: *diet, exercise, mental health, health literacy*, or *not related to pregnancy*. The two-step approach identified articles in two steps. The first stage classified articles based on whether or not they are related to pregnancy. Then, the second step classified a pregnancy-relevant article into four topics: *diet, exercise, mental health*, and *health literacy*.

#### 2.2.1 Experimental settings

In this study, we used 5-fold cross-validation to obtain a distribution of the experimental metrics and reported the macro-averaged precision (P), recall (R), and F1-scores (F). In each of the five folds, we used one-fold (20%) as the hold-out test set and the remaining four folds (80%) as the training set. We leveraged the state-of-the-art Bidirectional Encoder Representations from Transformers [BERT (30)] and further fine-tuned the pertained model in downstream tasks. The model took a sequence of tokens with a maximum length of 512 and produced a 768-dimensional sequence representation vector. For text that is shorter than 512 tokens, we added paddings (empty tokens) to the end of the text to make up the length. For text that is longer than 512 tokens, we used the first 512 tokens as the input. Then, two fully connected layers are appended on top of the pooler output layer of the BERT model. Finally, a SoftMax layer is used to map the representation vector to the target label space. For the BERT model, we fine-tuned the “BioBERT” (31) model with the training data for 20 epochs with a learning rate of 2*10^−5^ and batch size of 16. We adopted AdamW (32) as the optimizer and cross-entropy as the loss function. All BERT models were constructed on Amazon SageMaker with an NVIDIA T4 GPU with 128 GB of GPU memory. Pytorch (1.12.1) library was used to develop BERT models.

We compared the BERT model with the Generative Pre-trained Transformer model (GPT). We used the Dec 15, 2022 version of gpt-3.5-turbo to classify articles by question answering. Different questions corresponded to different classification methods and article types. For one-step approach, we asked gpt-3.5-turbo the question “Only give a one-word answer for which category this text belongs to: *pregnancy-related diet, pregnancy-related exercise, pregnancy-related mental health, pregnancy-related health literacy*, or *pregnancy-unrelated*.” This allowed gpt-3.5-turbo to directly classify the articles into one of five categories. For the two-step method and the classification of pregnancy-related and pregnancy-topics, we first used the question “Only answer yes or no to the question: Is the following text related to pregnancy and not” to classify the articles as pregnancy-related or not. Further, for the pregnancy-related articles, we asked the question “Only give a one-word answer for which category this text belongs to: *diet, exercise, mental health*, or *health literacy*” to distinguish the pregnancy topics. We also compared the proposed BERT models with traditional machine learning methods. In particular, we experimented with Random Forests (RF) using TF-IDF features. We set the word frequency to be (< 4) and capped the dimensionality to 1500 features. The number of trees in the forest was 200. Scikit-learn (1.0.2) library was used for the RF model.

## 3 Results

### 3.1 Overall performance

Table 2 shows the performance of RF, gpt-3.5-turbo, and BERT-based classifications using one-step and two-step approaches. In the case of the one-step approach, we observed that BERT obtained the best performance with a macro-averaged precision of 0.957, a recall of 0.951, and an F1-score of 0.953, which are 4.01, 8.60, and 6.50% higher than those of RF, and 18.6, 4.6, and 14.9% higher than those of gpt-3.5-turbo, respectively. The BERT-based two-step approach achieved micro-averaged precision, recall, and F1-score of 0.919, 0.926, and 0.921, respectively. The RF-based two-step approach achieved macro-averaged precision, recall, and F1-score of 0.888, 0.902, and 0.892, respectively. The gpt-3.5-turbo-based two-step approach obtained macro-averaged precision, recall, and F1-score of 0.876, 0.922, and 0.890, respectively. When comparing the end-to-end model with the two-step approach, we found that the end-to-end BERT model performed better than the two-step BERT-based approach. However, the end-to-end gpt-3.5-turbo model is worse than its counterparts.

**Table 2:** Overall performances of RFs, gpt-3.5-turbo, and BERT models.

		**Random forests**			**gpt-3.5-turbo**			**BERT**		
**Models**	**Categories**	**P**	**R**	**F**	**P**	**R**	**F**	**P**	**R**	**F**
One-step (end-to-end)	Diet	0.910	0.919	0.915	0.793	0.970	0.873	0.981	0.966	0.973
	Exercise	0.946	0.906	0.926	0.711	1.000	0.831	0.953	0.979	0.965
	Mental health	0.916	0.784	0.844	0.485	0.979	0.648	0.951	0.943	0.945
	Health literacy	0.914	0.740	0.818	0.867	0.980	0.920	0.932	0.914	0.921
	Unrelated	0.902	0.977	0.938	1.000	0.599	0.749	0.970	0.954	0.962
	*Macro*	*0.917*	*0.865*	*0.888*	*0.771*	*0.906*	*0.804*	*0.957*	*0.951*	*0.953*
Two-step	Diet	0.876	0.928	0.899	0.932	0.970	0.951	0.918	0.971	0.943
	Exercise	0.890	0.938	0.909	0.795	0.969	0.873	0.910	0.953	0.927
	Mental health	0.832	0.889	0.856	0.688	0.979	0.808	0.888	0.897	0.892
	Health literacy	0.884	0.820	0.849	1.000	0.840	0.913	0.923	0.873	0.895
	Unrelated	0.957	0.937	0.947	0.966	0.854	0.906	0.957	0.937	0.947
	*Macro*	*0.888*	*0.902*	*0.892*	*0.876*	*0.922*	*0.890*	*0.919*	*0.926*	*0.921*

P, Precision; R, Recall; F, F1-score.

The macro-averaged values are italicized and the highest F1-scores are underlined.

### 3.2 Classification by relevance to pregnancy

**Figure 2:**
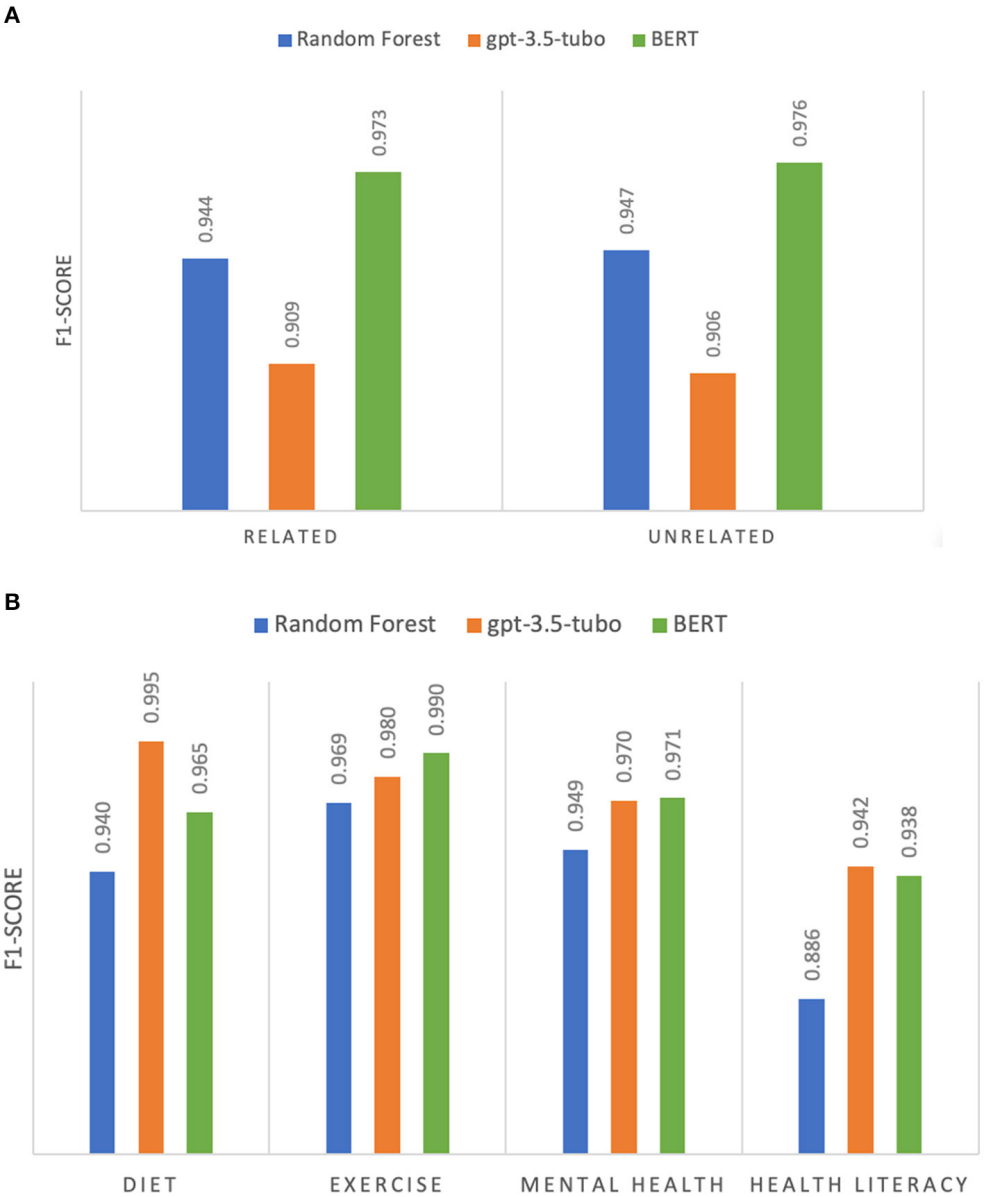
**(A)** F1-scores of the classification of pregnancy-relevant articles. **(B)** F1-scores of the classification of pregnancy topics.

Figure 2 and Supplementary Table S1 show the binary classification performance comparison between RF, gpt-3.5-turbo, and BERT. We observed that BERT achieved the best performance with macro-averaged precision, recall, and F1-score of 0.975, 0.974, and 0.974, respectively for classifying pregnancy-related and unrelated articles. Detailed counts of BERT-based model such as true positive, false positive, and false negative of each fold can be found in Supplementary Table S2. RF and gpt-3.5-turbo showed lower F1-scores of 0.945 and 0.908 than BERT.

### 3.3 Classification by pregnancy topics

Figure 2 and Supplementary Table S3 show that the gpt-3.5-turbo model achieved the best performance with macro-averaged precision, recall, and F1-score of 0.973, 0.972, and 0.972, respectively. Detailed counts of gpt-3.5-turbo model such as the true positive, false positive, and false negative of each fold can be found in Supplementary Table S4. The performance of gpt-3.5-turbo was 3.53, 3.43, and 3.53% higher than that of RF and 0.52, 0.48, and 0.56% higher than that of BERT.

## 4 Discussion

Our pipelines demonstrated the ability of the NLP algorithm to identify pregnancy-related online health education materials. The end-to-end BERT model obtained better results than the two-step BERT by 3.22% in the F1-score. For RF, although the two-step approach increased the recall by 3.74%, the precision decreased by 2.95%. Similarly, the two-step approach using gpt-3.5-turbo increased the F1-score by 8.6%. BERT outperformed RF and gpt-3.5-turbo in all scenarios. While end-to-end had better performance than two-step, it lacks interpretability and requires retraining when new topics are added. In comparison, in the two-step approach, we only need to train the second one and there is flexibility in adding additional topics for articles.

In the binary classification of pregnancy-related articles, RF obtained a satisfactory F1-score. The performance of BERT in this task is not an obvious advantage; although it improved by 2.9% it required more time and computing cost. In addition, the type of input text had little effect on the performance of the model, and the title achieved high accuracy under extremely fast computation speed. In the classification of pregnancy topics, the performance advantage of BERT and gpt-3.5-turbo are significant. Despite the demonstrated performance of gpt-3.5-turbo in general question answering, we discovered that, due to the specialized needs of our questioning (related to pregnancy but does not mention postpartum depression), we needed a dedicated NLP model for our use case. Still, gpt-3.5-turbo demonstrated superior performance in pregnancy topic classification, thus showing its potential for use in the future.

We performed our experiments using 392 pregnancy-related articles, and the BERT-based system performed better in most cases. In the future, we plan to add more articles with additional categories related to pregnancy. We searched the articles for our experiments manually, and it is possible that our search did not retrieve all relevant articles. We plan to implement automated web crawlers to collect the articles from the identified sources more efficiently in the future.

### 4.1 Limitations

Here are two limitations of our study. First, we intentionally focused on articles from 2019 onwards as the gold standard to capture recent trends and understandings in health topics. While we acknowledge the potential relevance and value of articles published prior to 2019, our current model was designed with a specific timeframe in mind to ensure contemporary relevance. However, we will consider expanding our dataset to include older articles in future iterations or expansions of the model. Second, the BERT-based system has a limitation of accepting only 512 tokens as input from each document. One potential solution to this challenge is to divide the document into multiple chunks and feed them individually to the BERT-based system. However, since the system already outperformed all other systems in terms of performance, we decided not to pursue this approach in order to avoid complicating the development process.

## 5 Conclusion

Given the abundance of high-quality lay articles and the knowledge- and labor-intensiveness of creating patient education materials, we proposed that lay articles related to pregnancy could be categorized and repurposed into educational and communicational materials for patients through NLP models. We explored state-of-the-art BERT models and gpt-3.5-turbo in an effort to obtain superior performances in classifying these articles. Current work is underway to incorporate the model based on gpt-3.5-turbo into our research website to scale the volume and diversity of content the website can provide to patients with diverse backgrounds and needs. Future work involves an on-going pilot evaluation with patients to assess acceptability and technical feasibility.

## Data availability statement

The original contributions presented in the study are publicly available. This data can be found here: https://github.com/brajagopalcse/AI_Driven_Patient_Education_Materials.

## Author contributions

BP: Conceptualization, Formal analysis, Investigation, Methodology, Software, Supervision, Validation, Writing—original draft, Writing—review & editing, Data curation, Resources, Visualization. ZS: Data curation, Formal analysis, Software, Writing—review & editing, Validation. ZC: Data curation, Formal analysis, Software, Writing—review & editing, Validation. PK: Data curation, Formal analysis, Methodology, Project administration, Software, Validation, Writing—review & editing. AA: Data curation, Formal analysis, Writing—review & editing. YL: Data curation, Formal analysis, Software, Writing—review & editing, Validation. RJ: Conceptualization, Data curation, Writing—review & editing. CJ: Conceptualization, Data curation, Writing—review & editing. DD: Conceptualization, Data curation, Writing—review & editing. JP: Writing—review & editing, Supervision. YP: Investigation, Writing—review & editing, Supervision. YZ: Conceptualization, Formal analysis, Funding acquisition, Investigation, Methodology, Project administration, Resources, Supervision, Visualization, Writing—original draft, Writing—review & editing, Data curation, Validation.
