# Supplementary material for: Automated classification of lay health articles using natural language processing: a case study on pregnancy health and postpartum depression
Source: Front Psychiatry. 2023 Nov 20;14:1258887. doi: 10.3389/fpsyt.2023.1258887 (PMC10694448; doi:10.3389/fpsyt.2023.1258887)
Supplement: Supplementary file 1 [file Data_Sheet_1.pdf]

SUPPLEMENTARY MATERIAL

The dataset and code used in this study can be found at:
https://github.com/YiyeZhangLab/AI_Driven_Patient_Education_Materials

Table S1. Performances of classification of pregnancy-relevant articles. P: Precision; R: Recall; F: F1-score

	**Random Forests**			**gpt-3.5-turbo**			**BERT**		
**Categories**	**P**	**R**	**F**	**P**	**R**	**F**	**P**	**R**	**F**
**Related**	0.934	0.954	0.944	0.858	0.967	0.909	0.979	0.967	0.973
**Unrelated**	0.957	0.937	0.947	0.966	0.854	0.906	0.971	0.982	0.976
macro	*0.946*	*0.946*	***0.945***	*0.912*	*0.910*	***0.908***	*0.975*	*0.974*	***0.974***

Table S2. The detailed performance of pregnancy-relevant articles classification using BERT. TP:true positive; FP:false positive; FN:false negative

**Fold**	**Categories**	**TP**	**FP**	**FN**	**P**	**R**	**F**
1	**Related**	78	0	4	1.000	0.951	0.975
	**Unrelated**	83	4	0	0.954	1.000	0.976
2	**Related**	76	2	0	0.974	1.000	0.987
	**Unrelated**	87	0	2	1.000	0.978	0.989
3	**Related**	74	2	2	0.974	0.974	0.974
	**Unrelated**	87	2	2	0.978	0.978	0.978
4	**Related**	71	3	6	0.960	0.922	0.940
	**Unrelated**	84	6	3	0.933	0.966	0.949
5	**Related**	80	1	1	0.988	0.988	0.988
	**Unrelated**	82	1	1	0.988	0.988	0.988
**macro**					0.975	0.974	0.974

Table S3. Performances of classification of pregnancy topics.

	**Random Forests**			**gpt-3.5-turbo**			**BERT**		
**Categories**	**P**	**R**	**F**	**P**	**R**	**F**	**P**	**R**	**F**
**Diet**	0.919	0.965	0.940	0.990	1.000	0.995	0.943	0.991	0.965
**Exercise**	0.966	0.973	0.969	0.960	1.000	0.980	0.992	0.988	0.990
**Mental Health**	0.944	0.958	0.949	0.942	1.000	0.970	1.000	0.946	0.971
**Health Literacy**	0.922	0.857	0.886	1.000	0.890	0.942	0.936	0.946	0.938
macro	*0.938*	*0.938*	***0.936***	*0.973*	*0.972*	***0.972***	*0.968*	*0.968*	***0.966***

Table S4. The detailed performance of pregnancy articles classification using gpt-3.5-turbo

**Categories**	**TP**	**FP**	**FN**	**P**	**R**	**F**
**Diet**	99	1	0	0.990	1.000	0.995
**Exercise**	96	4	0	0.960	1.000	0.980
**Mental Health**	97	6	0	0.942	1.000	0.970
**Health Literacy**	89	0	11	1.000	0.890	0.942
**macro**				0.973	0.972	0.972

Table S5. A list of topics for unrelated categories

Cars, Cruises, Travel
General diet, General fitness, General mental health
Women’s exercise (not pregnancy)
Women’s mental health (not pregnancy)
Women’s diet (not pregnancy)
Men’s diet, Men’s fitness, Men’s mental health
